# Laparoscopic Right Adrenalectomy with Venous Tumor Thrombus from Contralateral Renal Cell Carcinoma Metastasis

**DOI:** 10.5152/tud.2023.23022

**Published:** 2023-07-01

**Authors:** Andreia Cardoso, Sara Anacleto, Catarina Laranjo Tinoco, João Pimentel Torres, Carlos Oliveira, Emanuel Carvalho-Dias

**Affiliations:** 1 Department of Urology, Hospital de Braga, Braga, Portugal; 2 University of Minho, School of Medicine, Braga, Portugal

## Objective

Renal cell carcinoma (RCC) cure is almost only possible through complete surgical tumor excision. For metastasis, complete metastasectomy is also the best therapeutic option available.^1^ Renal cell carcinoma adrenal metastases are rare, especially contralaterally,^2^ with adrenal vein tumor thrombus (AVTT) being exceedingly rare.^3,4^

Thus, we aim to present our technique for laparoscopic right adrenalectomy, in a very rare case report, of a contralateral RCC metastasis with AVTT.

## Methods

At 30 months of follow-up after left radical nephrectomy for low-risk clear cell RCC, a CT scan revealed a suspicious 26 × 20 mm lesion on the right adrenal. Thus, due to metachronous single RCC metastasis to the contralateral adrenal, complete metastasectomy, through right adrenalectomy, was proposed. Patient written informed consent was obtained.

## Results

We demonstrate our slightly modified and particular 5-port placement (without standard triangulation), operative team positioning, and surgical technique.

We place the first 10 mm trocar for the camera, just parallel to the rectus abdominis muscle, close to the umbilicus level, with the open Hassan technique. After pneumoperitoneum set at 12 mmHg, we place one 5 mm and one 10 mm trocars under direct vision, in the same straight line, for the main surgeon instruments. After cranial liver mobilization, we place another 5 mm port for liver retraction, in the same straight line, near xiphoid appendix. The last 5 mm trocar for the assistant is placed near the anterosuperior iliac spine, within the camera line.

We reflect the right colon until the pelvis, always following the avascular plane between the mesocolon and Gerota’s fascia, and then proceed with duodenum reflection (Kocher maneuver). We believe that especially in right adrenalectomy, it is important to gain adequate space to work in.

We dissect just anteriorly to the inferior vena cava (IVC) and identify the right renal vein and the protruding right adrenal gland. We start by completely releasing the adrenal gland from its surroundings, incising renal fascia, and dissecting the adrenal from the right renal vein, IVC, and kidney.

After careful adrenal dissection, we carefully look for an adrenal vein draining to the IVC, in which we found an apparent protruding AVTT. Using simple forceps, traction, and countertraction, we milked it away from the IVC to assure safe adrenal vein ligation and complete tumor excision, before Hem-O-Lok placement.

The operative time was 80 minutes. The pathologic report confirmed a 3.2 × 2.5 × 2.0 cm RCC metastasis. After 6 months, there is no recurrence nor complications.

## Conclusion

Adrenalectomy requires extreme care due to adrenals location, especially on the right. Venous thrombus demands additional skills for its milking from the IVC.^3-5^

Some can argue that the vein should be first clipped and only afterward the adrenal be released. However, the contrary allows better vein access and control.

In our experience, our straight-line port-positioning enhances ergonomics and access and visualization of the surgical field, so we usually use it for kidney, adrenal, and upper urinary tract procedures.

Video see link: https://doi.org/10.5152/tud.2023.23022

### Informed Consent:

Written informed consent was obtained from the patient who agreed to take part in the study.

### Peer-review:

Externally peer-reviewed.

### Author Contributions:

Concept – C.O., E.C.D.; Design – A.C., E.C.D.; Supervision – J.P.T., E.C.D.; Resources – A.C., E.C.D.; Materials – S.A., E.C.D.; Data Collection and/or Processing – A.C., E.C.D.; Analysis and/or Interpretation – A.C., E.C.D.; Literature Search – A.C., C.T.; Writing – A.C.; Critical Review – E.C.D. 

### Declaration of Interests:

The authors have no conflict of interest to declare.

### Funding:

The authors declared that this study has received no financial support.

